# Evaluation and pilot implementation of essential interventions for the management of hypertension and prevention of cardiovascular diseases in primary health care in the Republic of Tajikistan

**DOI:** 10.12688/f1000research.20234.1

**Published:** 2019-09-13

**Authors:** Dylan R.J. Collins, Tiina Laatikainen, Mekhri Shoismatuloeva, Isfandiyor Mahmudzoha, Zakriya Rahimov, Dilorom Sultonova, Bunafsha Jonova, Jill L. Farrington

**Affiliations:** 1 University of British Columbia, Vancouver, Canada; 2 Institute of Public Health and Clinical Nutrition, Helsinki, Finland; 3 World Health Organization Regional Office for Europe, Copenhagen, Denmark; 4 Ministry of Health and Social Protection, Dushanbe, Tajikistan; 5 Service of State Supervision for Medical Activities and Social Protection of the Population of the Republic of Tajikistan, Dushanbe, Tajikistan; 6 Republican Clinical and Training Centre of Family Medicine, Dushanbe, Tajikistan

**Keywords:** Cardiovascular disease, Tajikistan, Hypertension, Public Health

## Abstract

**Background:** Non-communicable diseases (NCDs) are the leading cause of death worldwide and are a major burden in Tajikistan. The health system of Tajikistan is still shaped by the country's Soviet legacy and the pace of reform has been slow, with high patient out-of-pocket expenditure. The aim of this study is to determine the feasibility of implementing and evaluating essential interventions for the management of hypertension and prevention of cardiovascular disease in primary health care in Tajikistan.

**Methods and analysis:** A pragmatic, sequential mixed methods explanatory design, composed of quantitative and qualitative strands will be used with greater weighting of the quantitative strand. A single geographic district was nominated by the Ministry of Health and chosen for implementation. All primary health care centres in the district that meet inclusion criteria will be included; half will be randomly assigned to the intervention arm and half to the control arm. The overall process is organized into seven steps: (1) refresh clinical decision-making tools including open source WHO PEN and HEARTS resources; (2) update training package for primary health care workers; (3) collection of baseline data; (4) training staff in intervention clinics; (5) implementation of protocols and implementation coaching; (6) collection of follow-up data after 12 months; (7) evaluation of results and sharing experience.

**Ethics and dissemination: **Ethical review and approval have been obtained. Findings will be disseminated at the participant level, national level through a national conference of key stakeholders, and internationally through publication in an open-access peer review journal.

## Introduction

Non-communicable diseases (NCDs) are the leading cause of death worldwide, causing 41 million deaths in 2016 ^1^. Half were due to cardiovascular diseases (CVD) ^2^. Primary health care (PHC) systems are an essential component required to tackle this global burden ^3^. While strong PHC systems based on the principles of family medicine contribute to achieving universal health coverage, better health outcomes and economic and social development, many nations lack PHC capacity ^4, 5^. To help national governments develop primary health care capacity for NCDs, the World Health Assembly endorsed the WHO Global Action Plan for the Prevention and Control of NCDs 2013–2020 ^6, 7^. 

A country of the former Soviet Union, Tajikistan is located in Central Asia; the capital city is Dushanbe. Ranking among the poorest in the WHO European Region, Tajikistan had a gross domestic product (GDP) of 796 USD per capita in 2016 ^8^. The WHO Regional Office for Europe continues to support Tajikistan to implement the Global Action Plan, including support for the development, testing, and national scale-up of evidence-based clinical guidance and health policy for the prevention and management of NCDs.

### Non-communicable diseases in Tajikistan

NCDs are a major burden in Tajikistan. The age-standardized NCD mortality rate was 685.3 per 100,000 in 2015 ^9^. In 2016, the probability of dying prematurely (between the ages of 30 and 70 years) from CVD, cancer, diabetes, or chronic respiratory disease was 25.3%; like most populations, this rate is higher for men than women ^10^.

The Ministry of Health of Tajikistan and the World Health Organization Regional Office for Europe recently completed a population STEPS survey (unpublished by WHO) which indicated that 25.7% of men are current users of tobacco (including smokeless tobacco) although rates are low amongst women (0.2%) ^11^. For cultural reasons, alcohol use is relatively low with only 9.4% of men and 0.2% of women identified as current drinkers, and lifetime abstention is very high. The prevalence of obesity is 11.9% in women and 15.4% in men, and around half (46.7%) the adult population are overweight.

The prevalence of raised blood pressure (BP) (defined as systolic BP ≥ 140 mmHg and/or diastolic BP ≥ 90 mmHg or currently taking medication for raised blood pressure) among the adult population is 32.2%. Less than half of men (44.3%) have never had their blood pressure measured; the same is true for 24.7% of women. Nearly all (96.9%) adults have never had cholesterol measured. One-in-seven (13.8%) people aged 40–69 years have a 10-year fatal or non-fatal CVD risk of over 30% (including those with an existing CVD).

### Primary health care in the Tajikistan

The health system of Tajikistan is still shaped by the country's Soviet legacy and the pace of reform has been slow ^12^. The country has the one of the lowest total health expenditure (THE) per capita in the WHO European Region, estimated to be 6.88% of GDP in 2014, and the proportion of THE that is financed privately through out-of-pocket payments (OOP) is 61.69%; second highest in the WHO European Region ^8^. Although formally free, OOP expenditure for PHC is common and expenditure on pharmaceuticals is the biggest financial burden.

Recent reforms have aimed to strengthen PHC, but success has been mixed. In 2010, Tajikistan launched the National Health Strategy for the period 2010–2020 ^13^, which recognized the importance of health system strengthening, and highlighted the development of PHC based on family medicine practice as a top priority. The National Programme on the Development of Family Medicine 2011–2015 in Tajikistan had the goal of ensuring the sustainable development of PHC according to the principles of family medicine. Some substantial improvements were achieved, including trainings for the health workforce, the review of clinical protocols, promotion of quality of care, increasing capacity in family medicine, improvements in evidence-based practice, and greater availability of resources. Nevertheless, challenges have been highlighted, including insufficient pace and scale of initiatives, and the scope of work and distribution of family doctors across the country ^14^. Although some computers have been installed in district health care facilities to introduce electronic submission of statistical reports, the vast majority of rural health clinics rely on paper records which need to be completed manually, hindering reliable data collection, processing and analysis ^12^.

In Tajikistan, health services are delivered at five levels: rural (village), district (rayon), city, oblast (regional), and republican (national). In villages, PHC services are provided in rural health centres (RHCs) with a family medicine doctor or health houses with feldshers, family medicine nurses and midwives.

### Essential interventions to manage hypertension and prevent CVD in primary health care

In order to build capacity in PHC and ultimately prevent premature mortality from major NCDs, from 2013 to 2015 Tajikistan endeavoured to adapt and pilot the World Health Organization Package of Essential NCD Intervention for Primary Healthcare in Low Resource Settings (WHO PEN) ^5^. WHO PEN includes simplified clinical protocols which together cover the integrated management of hypertension and diabetes, as well as education and counselling on healthy behaviours aimed to prevent CVD. The central strategy of this integrated approach is the use of total cardiovascular risk assessment to stratify and target individuals at high CVD risk, a process considered to be a “best buy” intervention by the WHO ^15^.

These early efforts led to limited success. A national PEN steering group was established in 2012, facilities were assessed for access to essential medicines and technologies, national clinical guidelines were updated to include the WHO/ISH risk prediction chart ^16^, and family doctors and nurses from 9 pilot facilities received two-day training on the WHO PEN protocols during 2013/14. Apart from a “refresh” training for selected clinics two years later, there was no further intervention, and the lack of systematic monitoring and evaluation made it difficult to demonstrate any significant change in clinical practice, disease detection, disease control rates or clinical outcomes.

In 2016, Tajikistan agreed to embark on a pilot of the global WHO HEARTS initiative supported by WHO ^17^. Work on this was able to proceed in earnest in 2018 with the publication of the HEARTS technical package which aimed to provide a strategic approach to improving cardiovascular health in countries ^18^. It comprises six modules and an implementation guide to support Ministries of Health to strengthen CVD management in primary health care settings.

Led by the Ministry of Health and Social Protection, the WHO PEN steering group was expanded to include chief specialists, district and rural health facility managers and clinicians, the Republican Clinical and Training Center of Family Medicine and the Service of State Supervision for Medical Activities and Social Protection of the Population of the Republic of Tajikistan. The national steering group is supported by an international team of experts coordinated jointly by the WHO Regional Office for Europe and the WHO Country Office in Tajikistan. Tajikistan has a particular interest in systems monitoring, and in developing sustainable and scalable approaches, building upon other initiatives in PHC reforms and quality improvement.

## Aim and objectives

### Aim

The aim is to determine the feasibility of implementing and evaluating essential interventions for the management of hypertension and prevention of CVD in PHC in Tajikistan.

### Objectives

In order to achieve this aim, the objectives are to:

1. Determine the baseline performance of PHC services with respect to essential interventions for the management of hypertension and prevention of CVD;
2. Assess the ability to conduct practical trainings for primary care doctors and nurses and implement Tajikistan-adapted HEARTS tools, in one pilot region of Tajikistan;
3. Estimate the change in clinical practice with respect to essential interventions for the management of hypertension and prevention of CVD after 12 months of implementation;
4. Determine the feasibility of collecting quantitative data required for future studies of effectiveness from routine clinical data.

## Methods and analysis

As part of a larger programme of implementation research, we have previously developed an approach to evaluating and piloting essential interventions for NCDs in low-resource settings, and have adapted this methodology to suit the context of Tajikistan ^19^.

### Overview of process and design

The general approach to developing, piloting, and evaluating essential interventions for the management of hypertension and prevention of CVD in PHC in Tajikistan are outlined in the following seven steps. A more detailed methodological description is presented in the section that follows.

***Step One: Refresh clinical decision-making tools***. Tajikistan national guidelines for PHC exist in a printed book format, and these books are available and used by family doctors. The national guidelines for hypertension (updated in 2018) endorse the use of WHO/ISH CVD risk charts, and some family doctors were previously trained on the use of WHO PEN. However, there is a lack of simple clinical decision support tools, such as laminated one-page algorithm cards for the treatment of hypertension. As such, the following tools will be adapted to be in-line with existing national guidelines, used for training, and disseminated to PHC workers in intervention clinics.

- HEARTS Hypertension Protocol – Diuretic as First-line Treatment
- HEARTS WHO PEN Diabetes Protocol
- WHO PEN Protocol 1 – adapted to integrate with HEARTS Hypertension Protocol
- WHO PEN Protocol 2 – adapted to integrated with HEARTS Hypertension Protocol
- Body-Mass Index Calculation Table
- WHO CVD Risk Charts – WHO/ISH no cholesterol charts
- Integrated Management of Hypertension and Diabetes Algorithm (HEARTS Risk Module)
- AUDIT and Fagerstrom Clinical Decision Support Tools

While the content of these tools is consistent with national guidelines, a ministerial order may be required to inform clinicians in the intervention clinics that it is appropriate to follow these updated versions of the already approved national guidelines.

***Step Two: Update training package for primary health care workers***. There is an existing two-day training developed for the implementation of WHO PEN in Tajikistan. This training package will be reviewed for overall content, pre- and post-training evaluation of participant knowledge, adult learning methods, practical exercises, consistency with refreshed clinical decision support tools and protocols, and assessment of practical clinical skills.

Furthermore, based on the previous experience of implementing WHO PEN in Tajikistan, the following topics have come to light as important to emphasize in the updated training package:

- Organization of care
- Task sharing between doctors and nurses
- Meaningful clinical record keeping
- Approach to managing a patient without laboratory cholesterol tests
- Appropriate and timely follow-up of patients
- Optimization of nurse home-visit tasks to include greater focus on underserved populations at risk for CVD (e.g. older men)
- Measuring inequalities in care and outcomes by gender
- Raising population awareness of cardiovascular diseases

***Step Three: Collection of baseline data***. Baseline data will be collected from all clinics in the included district before implementation (i.e. training) begins. Data for quantitative indicators will be extracted by randomly sampling individual paper-based patient records from all PHC units using a standardized data collection instrument. This will be done by a small team of specially-trained national experts. After analysis, each health clinic will receive a summary report of their facility’s performance with respect to key project indicators. This report will not be powered for statistical significance, but will be used as a quality improvement exercise to show how routine clinical data can be used to improve clinical practice, for example by setting improvement targets.

***Step Four: Training staff in intervention clinics***. All doctors and nurses from the PHC centres in the intervention arm will be invited to be trained together by a national team of experts in groups of approximately 30. It is estimated that up to 100 health workers will be trained in total. Given the human resources, the trainers will be themselves a group of family doctors and nurses, rather than narrow medical specialists or academics, so as to maintain direct relevancy to practical aspects of PHC services.

The training will be two-days; therefore it is estimated that two trainings can be conducted in one week (Monday/Tuesday and Thursday/Friday). Thus, 60 participants can be trained per week; two weeks will be required to train all doctors and nurses in the pilot district. A ministerial order will be required to relieve the health staff from their duties to attend the training.

***Step Five: Implementation of protocols and implementation coaching***. Trained participants from intervention clinics will then be free to implement the clinical protocols and change their clinical practice, without incentives, for 12 months. During this time, a team of national and district experts will be created to offer support (distance and on-the-job) to the PHC centres. These teams will visit each clinic once every three months (for a total of four visits) and use a set of standardized instruments to review performance and provide constructive and timely feedback to the health facility manager and individual clinicians. The tools used during the support visits and implementation coaching will include:

- HEARTS Treatment Supervision Form – with minor adaptations to local context
- HEARTS Patient Interview Report Card – with minor adaptations to local context
- HEARTS Summary of Supervision Visits – with minor adaptations to local context
- Structured Interview and Debrief Conversation Template – to be developed

In addition to clinic visits every three months, representatives from each clinic will be brought together after 6 months of implementation for a workshop. This will be an opportunity for the project management team to provide support based on the needs identified in clinic visits, to encourage target setting, quality improvement and peer support, as well as gather more information about implementation barriers.

***Step Six: Collection of follow-up data***. After 12 months, using the same method and data collection instruments used to collect baseline quantitative data (Step Three), data will again be extracted from randomly selected individual paper-based patient records from both intervention and control health centres. As was done at baseline, each health clinic will receive a summary report of their facility’s performance with respect to key project indicators comparing baseline to follow-up.

One-on-one semi structured interviews will be conducted with doctors, nurses, and health facility managers in the intervention clinics at 12 months follow-up, and these qualitative data will be analysed thematically for explanatory themes.

***Step Seven: Evaluation of results and sharing experience***. The findings of the quantitative and qualitative analyses will be integrated in a final report and shared with key stakeholders, including health staff from the participating primary health care centres. The results will also be shared at a national conference and in an open-access peer reviewed journal, in order to inform the future development of primary health care capacity of Tajikistan and other similar settings.

### Methodological design

**Figure 1.**
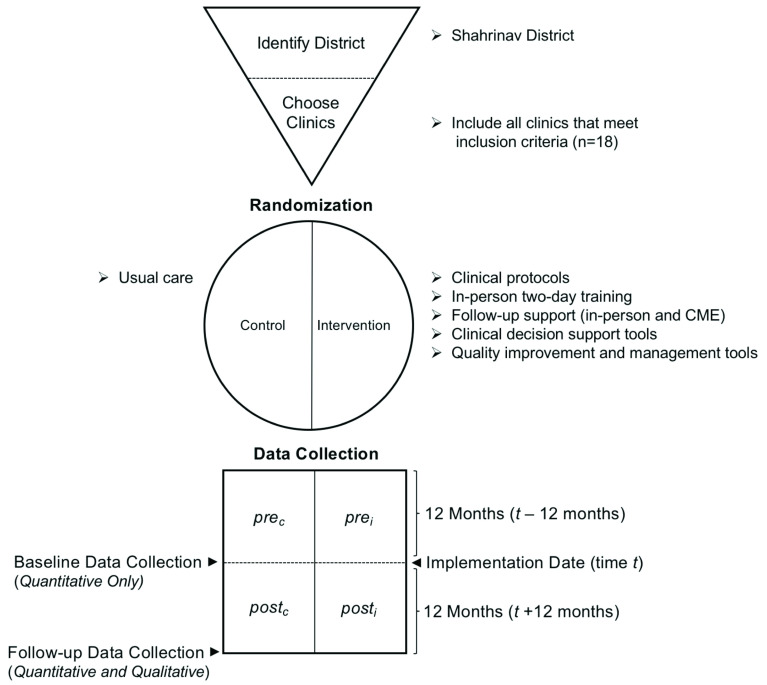
Illustration of the mixed methods evaluation design using the GATE frame structure ^20^.

A pragmatic, sequential mixed methods explanatory design, composed of quantitative and qualitative strands will be used ( Figure 1). The quantitative strand will be weighted more than the qualitative strand. A mixed methods design was chosen because it allows for the use of qualitative data to enlighten and explain the quantitative findings, but due to limited capacity to conduct qualitative research in Tajikistan, the qualitative strand will be weighted less than the quantitative strand.

Due to resource constraints, and several ongoing PHC reform projects, a single district was nominated by the Ministry of Health and chosen for implementation (rather than multiple districts). All PHC in the district that meet inclusion criteria will be included; half will be randomly assigned to the intervention arm and half to the control arm. Baseline data will be collected from all clinics in the intervention arm and the control arm using the same standardized data extraction form.

### Selection of primary health care centres

**Figure 2.**
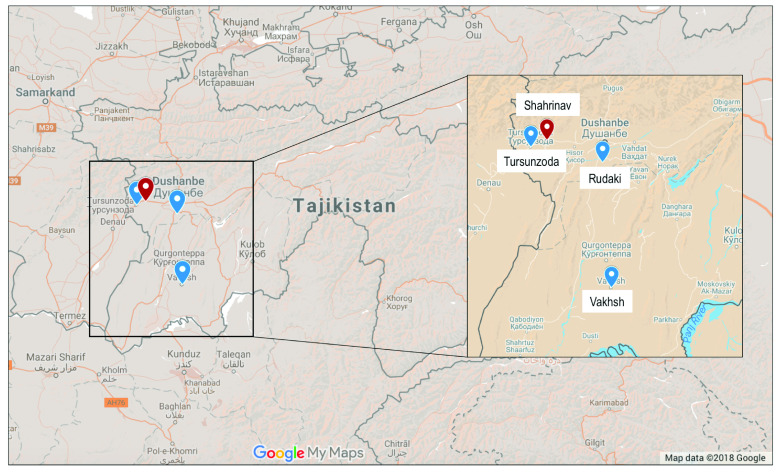
Map of Tajikistan indicating the four pilot areas considered. Shahrinav is indicated in red.

The national working group initially nominated four districts for consideration which had participated in other projects: while three of these are subordinate districts (“Rayons”) of Dushanbe, the 4 ^th^ (Vakhsh) is nearby to Dushanbe in the Oblast of Khatlon ( Figure 2).

In order to provide more intensive support and ensure the quality of data collection, it was decided to focus on only one district. All PHC centres and the associated family doctors and nurses will be invited to take part in the training and pilot implementation. Shahrinav was chosen after considering its size, exposure to previous interventions, proximity to Dushanbe, year-round road access, and by recommendation from the Ministry of Health.

Within Shahrinav district, all PHCs were compared against the following exclusion criteria: (1) City Health Centre designation; (2) presence of narrow specialists (i.e. any physicians with specialization other than family medicine) within the clinic; (3) clinics with a family doctor to patient ratio of 1:4000 or more; or (4) clinics where information on the number of family doctors and/or family doctor to patient ratio is not available. If one or more of these four exclusion criteria were met, the clinic will not be included in the intervention or control arm.

### Comparison

Clinics allocated to the control arm will not receive the intervention; clinicians will proceed with usual care. The performance of individual health facilities will be compared from baseline to follow-up. To compare change in performance between intervention and control arms, the data from all intervention clinics will be aggregated together and compared to aggregated data of all control clinics.

### Quantitative indictors

Given the emphasis of hypertension management, including total CVD risk approach, indicators were developed to be calculated from a sample of hypertensive patients. These include indicators along the hypertension care pathway, including detection of hypertension, CVD risk assessment, risk factor modifying prescriptions, and BP control. Table 1 shows the primary and secondary indicators, the question the indicator seeks to answer, and the respective numerator and denominator definitions which will be used in the calculations. 

**Table 1.** Primary and secondary indicators, their numerators and denominators, and questions the indicators answer.

Question	Indicator	Numerator	Denominator	Sample source
**Primary indicator**				
Is the blood pressure of hypertensive patients controlled?	Proportion of hypertensive patients whose blood pressure is under control	Number of patients with confirmed hypertension who visited the clinic at least once in the last 12 months, whose last reading was controlled (SBP <140 and DBP <90 mmHg)	Number of patients with confirmed hypertension who visited the clinic at least once in the last 12 months	Hypertension register
**Secondary indicators**				
Are patients with hypertension diagnosed and recorded on the hypertension register?	Proportion of the adult clinic catchment population who appear on the hypertension register	Number of adult patients who appear on the hypertension register	Expected number of hypertensive patients using the STEPS prevalence and size of clinic catchment population	Hypertension register
Are newly diagnosed hypertensive patients receiving timely and effective treatment?	Proportion of patients registered for hypertensive treatment at the health facility whose blood pressure is controlled 6 months after treatment initiation	Number of patients with controlled blood pressure (SBP <140 and DBP <90 mmHg) at the last clinic visit in the most recent quarter	Number of patients registered for treatment of hypertension during the quarter that ended 6 months previously	Hypertension register
Are key risk factors being measured amongst hypertensive patients?	Proportion of hypertensive patients who have all risk factor values recorded as required for calculation of a WHO PEN risk score	Number of patients aged 40 or older who have visited in the last 12 months who have all measurements required for calculation of a WHO PEN risk score within 12 months of the most recent date of visit	Number of hypertensive patients aged 40 or older who have visited in the last 12 months	Hypertension register
Are hypertensive patients being risk scored?	Proportion of hypertensive patients with a documented WHO PEN risk score	Number of hypertensive patients aged 40 or older who have visited in the last 12 months with a documented WHO PEN risk score	Number of hypertensive patients aged 40 or older who have visited in the last 12 months	Hypertension register
Are risk scores calculated correctly?	Proportion of hypertensive patients with a documented WHO PEN risk score that is correct	Number of hypertensive patients aged 40 or older who have visited in the last 12 months who have all measurements required for calculation of risk score within 12 months of the most recent date of visit, that have a documented risk score, that have a documented risk score that is correct	Number of hypertensive patients aged 40 or older who have visited in the last 12 months who have all measurements required for calculation of risk score within 12 months of the most recent date of visit, that have a documented risk score	Hypertension register
Is the blood pressure of high risk patients controlled?	Proportion of hypertensive patients with a WHO PEN risk score of > =30%, whose blood pressure is controlled	Patients with a true risk score of >=30% whose last documented blood pressure reading was controlled (SBP <130 and DBP 80 mmHg)	Patients with a true risk score of >=30%	Hypertension register
Is the blood pressure of lower risk patients controlled?	Proportion of hypertensive patients with a WHO PEN risk score of <30%, whose blood pressure is controlled	Patients with a true risk score of <30% whose last documented blood pressure reading was controlled (SBP <140 and DBP 90 mmHg)	Patients with a true risk score of <30%	Hypertension register
Are patients with existing disease, who do not require the calculation of a risk score to prescribe statins, prescribed statins?	Proportion of patients with existing CVD prescribed a statin	Number of patients with existing CVD prescribed a statin	Number of patients with existing CVD	Hypertension register
Are patients with existing CVD prescribed basic medications to reduce risk?	Proportion of patients with existing CVD prescribed a statin and aspirin and blood pressure lowering treatment	Number of patients with existing CVD prescribed a statin and aspirin and blood pressure lowering treatment	Number of patients with existing CVD	Hypertension register
Are statins prescribed correctly based on documented risk score?	Proportion of patients eligible based on documented risk score prescribed a statin	Number of patients with a WHO/ ISH risk of ≥30% documented in their chart prescribed a statin	Number of patients with a WHO/ISH risk of ≥30% documented in their chart	Hypertension register
Is the blood glucose of hypertensive patients with diabetes controlled?	Proportion of hypertensive patients with diabetes who have achieved glycaemic control as defined by last HbA1c measurements	Number of patients with diabetes 2 whose last HbA1c measurement was below personal target as defined by Moldova adapted WHO PEN 1	Number of patients with diabetes type 2	Hypertension register

### Data collection and management

***Quantitative data collection tool***. We developed a standardized data collection tool to collect the anonymized data required for calculation of each indicator ( Table 2). This tool will also be digitized such that it can be used on a computer (offline) or smartphone (online). We estimate it take 15 minutes per unique health record in order to extract data. Since there is a lack of electricity and mobile phone reception in many of the clinics, it is likely that data will have to be extracted first on paper and then later input to a database using an online data form. Data extractors will be blinded to the allocation of health care facilities to intervention or control for baseline data collection, but blinding is not possible for follow-up data collection because after the intervention period, allocation will be apparent when data collectors visit clinics and look at patient records.

**Table 2.** Standardized data collection form used to extract data from individual patient records.

Data collection question	Answer
What is your name? (Name of person extracting data)	
Date of Data Extraction (MM-DD-YYYY)	
Write the Clinic Name	
Is this a duplicate extraction?	
If it is a duplicate extraction, enter the number you and your extraction partner have assigned to this file.	
Date of Birth (MM-DD-YYYY)	
Sex (M/F)	
Smoking Status (Y/N)	
Diagnosis of Hypertension (Y/N)	
Date of Hypertension Diagnosis (MM-DD-YYYY)	
Can you find one or more blood pressure readings? (Y/N)	
Most Recent Systolic Blood Pressure	
Most Recent Diastolic Blood Pressure	
Date of the Most Recent Blood Pressure Measurement (MM-DD-YYYY)	
Diagnosis of Diabetes (Type 1, Type 2, No)	
Can you find one or more HbA1c measurements? (Y/N)	
Most recent HbA1c reading (mmol/mol)	
Date of the most recent HbA1c measurement? (MM-DD-YYYY)	
Can you find one or more total cholesterol measurements? (Y/N)	
Most recent total cholesterol reading (mmol/L)	
Date of the most recent cholesterol reading (MM-DD-YYYY)	
Was the patient prescribed a statin? (Y/N)	
What was the date of the statin prescription? (MM-DD-YYYY)	
What was the drug and dose?	
Does the patient have existing CVD? (Y/N)	
State the type of CVD	
Has the patient been prescribed acetylsalicylic acid (ASA or aspirin)? (Y/N)	
What was the most recent date that ASA was prescribed? (MM-DD-YYYY)	
Has the patient been prescribed anti-hypertensives? (Y/N)	
What was the most recent date that anti-hypertensives were prescribed? (MM-DD-YYYY)	
Can you find a documented WHO/ISH risk score? (Y/N)	
Enter the most recent documented WHO/ISH risk score (%)	
What was the date the risk score was documented? (MM-DD-YYYY)	
Please record any important notes about the data extraction here. Examples include an error you think may have been made, clarification of the units for measurements (e.g. mmol/L vs mg/dL). Or notes that you would like for yourself.	

**Figure 3.**
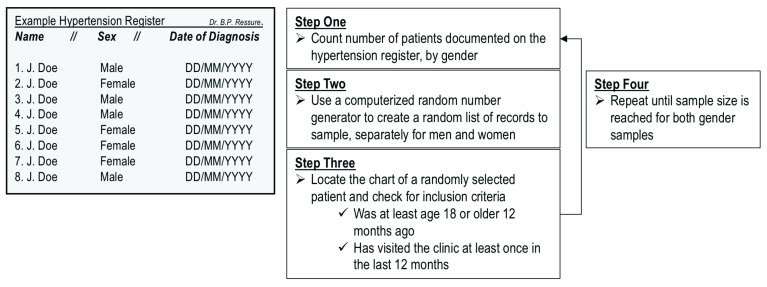
Illustration of the method used to randomly sample patient records from the hypertension register.

***Method of randomly sampling patient records***. Each family doctor owns and maintains a single register (list of patients) for hypertensive patients (henceforth “hypertension register”). The hypertension register will be used to randomly select patient records. A computerized random number generator will be used to randomly select patients from the register. The selected patient chart will then be found and checked to see if it meets two inclusion criteria: (1) the patient must have visited the clinic at least once in the previous 12 months; and (2) the patient must have been 18 years or older 12 months prior to the date of data selection. If both criteria are met, the record will be used for data extraction. If the record does not meet inclusion criteria, it will be returned and a new record will be randomly selected. This process will be repeated until the sample size for each family doctor has been met ( Figure 3).

***Sample size***. A total of 400 patient records will be sampled at baseline and follow-up from each of the intervention and control arms (n=800). The sample is based on a power calculation for the primary indicator (proportion of hypertensive patients whose blood pressure is controlled), estimated based on a WHO report that the control level in low and middle income countries is generally about 20–25% ^13^. A 10% point difference between the intervention and control arms could be detected having 310 to 350 observations in each group using 0.05 type I error rate and 0.2 type II error rate. Sampling will be stratified by gender and selected in a 1:1 ratio of men to women (200 men and 200 women per arm). In the event that there are too few patients of a given gender, patients from the opposite gender will be substituted.

***Data analysis***. The change in indicators from baseline to follow-up will be calculated. Subgroup analysis by age, gender, and other demographic features may be done as deemed appropriate.

***Qualitative data collection***.

**Semi-structured interviews** A purposive maximum variation sample of doctors and nurses from the intervention clinics will be invited to take part in semi-structured interviews at the end of the 12-month implementation phase. All participants will be required to provide written informed consent. Using an interview guide that will be adapted from previously published work ^21, 22^, interviews will be conducted one-on-one by members of the research team, audio recorded, and transcribed verbatim. Transcripts will be analysed thematically using framework thematic analysis, by two or more members of the research team, with oversight from an experienced qualitative researcher ^23^.

**Patient and public involvement** Neither patients nor the public were involved in the methodological design.

## Strengths and limitations of this study

- We report methodology to adapt, pilot, and evaluate the WHO HEARTS technical package in a low-resource setting in a low-income country that can be used as an example for other jurisdictions with a high burden of cardiovascular diseases
- Our methods focus on evaluating the effect of the WHO HEARTS interventions in a real-world health system and therefore are expected to yield practical information to inform the national scale up of essential interventions for non-communicable diseases
- The sample size is based on one primary indicator and it is therefore possible that some of the secondary indicators will be statistically underpowered
- Due to the practical and resource constraints, data collectors could not be blinded to follow-up data collection, and this could potentially bias the effect estimate
- Although an important stakeholder in implementation, patient perspectives were not included in the evaluation design

## Current study status

Data collection has been completed and analysis is underway.

## Ethics and dissemination

### Ethical review and approval

This project has been reviewed and granted ethical approval from the Republican Clinical Center for Family Medicine under the Ministry of Health and Social Protection of the Republic Tajikistan. As per the ethical approval review and the nature of this project, consent was not obtained from individual patients for their anonymized routine clinical data to be used.

### Dissemination of findings

Findings will be shared at the participant level (intervention clinics), national level through a national conference of key stakeholders, and internationally through publication in an open-access peer review journal. At the national level, the results will be used to assess the appropriateness and feasibility of national scale-up and mainstreaming into clinical practice. The raw data used in this project will not be made publicly available.

## Data availability

No data are associated with this article.

## References

[1] Benziger CP Roth GA Moran AE: The Global Burden of Disease Study and the Preventable Burden of NCD. *Glob Heart.* 2016;11(4):393–7. 10.1016/j.gheart.2016.10.024 27938824

[2] World Health Organization (WHO): World Health Statistics.2018 Reference Source

[3] World Health Organization (WHO): The World Health Report 2008 - Primary Health Care (Now More than Ever). Geneva;2008 Reference Source

[4] World Health Organization: The World Health Report - Health Systems Financing: the path to universal coverage. Geneva;2010 Reference Source 10.2471/BLT.10.078741 PMC2878164 20539847

[5] World Health Organization (WHO): Package of Essential Noncommunicable (PEN) disease interventions for primary health care in low-resource settings.2013 Reference Source

[6] United Nations: Political Declaration of the High-Level Meeting of the General Assembly on the Prevention and Control of Noncommunicable Diseaes. Resoluation A/RES/66/2. New York;2011 Reference Source

[7] World Health Organization (WHO): Global action plan for the prevention and control of NCDs 2013-2020.2013 Reference Source

[8] WHO Regional Office for Europe: European Health Information Gateway. Copenhagen;2018 Reference Source

[9] World Health Organization: Global Health Observatory - Total NCD Mortality by Country. Geneva;2018 Reference Source

[10] World Health Organization: Risk of Premature Death from the Four Target NCDs.2018 Reference Source

[11] WHO Regional Office for Europe: Understanding the health needs of Tajikistan.2018 Reference Source

[12] Khodjamurodov G Sodiqova D Akkazieva B: Tajikistan: Health System Review. *Health Syst Transit.* 2016;18(1):1–114. 27172509

[13] Ministry of Health and Social Protection of the Population of the Republic of Tajikistan: National Health Strategy of the Republic of Tajikistan 2010-2020. Dushanbe;2010 Reference Source

[14] WHO Regional Office for Europe: Review of the National Programme on the Development of Family Medicine 2011-2015 in Tajikistan. Copenhagen;2016 Reference Source

[15] World Health Organization: Tackling NCDs: “Best buys” and other recommended interventions for the prevention and control of noncommunicable diseases.2017 Reference Source

[16] World Health Organization: WHO/ISH Cardiovascular Risk Prediction Charts. Geneva;2007 Reference Source

[17] World Health Organization: Global HEARTS Initiative. Geneva;2018 Reference Source

[18] World Health Organization: HEARTS Technical Package. Geneva;2018 Reference Source

[19] Collins D Ciobanu A Laatikainen T: Protocol for the evaluation of a pilot implementation of essential interventions for the prevention of cardiovascular diseases in primary healthcare in the Republic of Moldova. *BMJ Open.* 2019;9(7):e025705. 10.1136/bmjopen-2018-025705 31278091 PMC6615880

[20] Jackson R Ameratunga S Broad J: The GATE frame: critical appraisal with pictures. *ACP journal club. United States;* 2006;144(2):A8–11. Reference Source 10.7326/ACPJC-2006-144-2-A08 16539343

[21] Collins DRJ Jobanputra K Frost T: Cardiovascular disease risk and prevention amongst Syrian refugees: mixed methods study of Médecins Sans Frontières programme in Jordan. *Confl Health.* 2017;11:14. 10.1186/s13031-017-0115-z 28725259 PMC5512828

[22] Liew SM Blacklock C Hislop J: Cardiovascular risk scores: qualitative study of how primary care practitioners understand and use them. *Br J Gen Pract.* 2013;63(611):e401–7. 10.3399/bjgp13X668195 23735411 PMC3662457

[23] Gale NK Heath G Cameron E: Using the framework method for the analysis of qualitative data in multi-disciplinary health research. *BMC Med Res Methodol.* 2013;13:117. 10.1186/1471-2288-13-117 24047204 PMC3848812

