# Supplementary material for: Evaluation and pilot implementation of essential interventions for the management of hypertension and prevention of cardiovascular diseases in primary health care in the Republic of Tajikistan
Source: F1000Res. 2019 Sep 13;8:1639. [Version 1] doi: 10.12688/f1000research.20234.1 (PMC7479501; doi:10.12688/f1000research.20234.1)
Supplement: Peer review history

# Peer review: Reviewer response for version 1

**DOI:** 10.5256/f1000research.22230.r66459

**Published:** 2020-09-08

**Authors:** Dagfinn Aune

**Affiliations:** 1 Department of Epidemiology and Biostatistics, Imperial College, London, UK

This is a study protocol for evaluation and pilot implementation of interventions for the management of hypertension and prevention of cardiovascular diseases in primary health care in Tajikistan. A pragmatic, sequential mixed methods explanatory design, composed of quantitative and qualitative strands will be used. A single geographic district was chosen for implementation and all primary care health care centers that meet inclusion criteria will be included. Half will be randomized to the intervention group and the other half to the control group. The overall process is organized in seven steps: refresh clinical decision-making tools – WHO PEN and HEARTS, update training package for health care workers, collection of baseline data, training staff in intervention clinics, implementation of protocols and implementation coaching, collection of follow-up data after 12 months, and evaluation of results and sharing experience.

 It would have been interesting to know a bit more about the data that will be collected. Will you collect clinical measurements (weight, height, waist measures, blood pressure) and take blood samples (cholesterol, glucose etc.)? Will you collect and data on dietary intake, smoking, physical activity and other lifestyle factors? Will there be any repeated measurements during the study follow-up?

 What exactly will the intervention entail?

 I see now some of the information is in Table 2. You might want to have more detailed data on smoking – including never, former and current smoking, cigarettes per day and duration, time since quitting in former smokers.

Is the study design appropriate for the research question?

Yes

Is the rationale for, and objectives of, the study clearly described?

Yes

Are sufficient details of the methods provided to allow replication by others?

Partly

Are the datasets clearly presented in a useable and accessible format?

Not applicable

Reviewer Expertise:

Epidemiology

I confirm that I have read this submission and believe that I have an appropriate level of expertise to confirm that it is of an acceptable scientific standard.

# Peer review: Reviewer response for version 1

**DOI:** 10.5256/f1000research.22230.r62985

**Published:** 2020-05-15

**Authors:** Anthony Etyang

**Affiliations:** 1 Department of Epidemiology and Demography, KEMRI Wellcome Trust Research Programme, Kilifi, Kenya

**General comments**

 Dylan Collins and colleagues present a manuscript outlining the implementation and plans for evaluation of essential interventions for the management of hypertension and prevention of cardiovascular diseases in primary health care in Tajikistan. This is an important and timely piece of work and the authors are to be commended for a very well written manuscript. The comments that I give below are very minor issues that would in my opinion improve the manuscript. I also raise some points for the authors to consider about the actual project recognizing that it might be too late for them to implement them.

**Specific Minor comments**

1. Title of the manuscript- shouldn’t implementation precede evaluation? Evaluation is the seventh step in the processes outlined.
2. Abstract- write out WHO PEN and HEARTS in full at first instance. 2 ^nd^ last word of abstract-should read ‘reviewed’.
3. Description of health system in Tajikistan having a Soviet legacy in both the abstract and main text-: This appears inappropriate and unnecessary to me especially when juxtaposed against sentences describing need for out of pocket payments which point to a capitalist type of health system, similar for example to that of the USA. Cuba is a communist state and has a well functioning health system (please see additional comment on this below).
4. P3, paragraph 4: Is the difference in the prevalence of obesity between men (15.4%) and women (11.9%) statistically significant? Please state if it is. I also find it unusual that the prevalence is higher in men than in women which is the opposite of the situation in most other countries. Are you able to offer any explanations for this?
5. Please explain what is meant by Soviet legacy in the health system. What specific aspects of the health system can be attributed to the fact that the country was part of the Soviet Union? As mentioned earlier, my feeling is that this reference to the past should be done away with unless a very clear and specific link can be shown.
6. It would be useful to provide background information on the numbers of health care workers in the country as well as Shahrinav district and the doctor/nurse to patient ratios.
7. Overview of process and design section (p4). Step 1: Refresh clinical decision making tools- apart from having laminated one-page algorithm cards for clinicians to use in the treatment of hypertension, has consideration been made to convert these into smart phone applications? Is this feasible in Tajikistan?
8. Please include more detail on how the random allocation of clinics to control and intervention arms was/will be conducted. Were representatives of all the clinics present to observe the randomization taking place?
9. Table 1: Is it possible to include magnitude of change in BP in mmHg as a secondary outcome? Proportion of controlled hypertensives is a rather crude measure.
10. Data analysis- This section needs to be expanded from the current two sentences as it will be critical to understanding the effect of the project. Is there a statistical analysis plan in place or is it being developed? For the primary outcome of proportion of hypertensives controlled, what test will be used and what confounders will be included in the model? Rather than comparing the change in indicators from baseline to follow up as the authors write, I would expect that the primary comparison would be proportion of controlled hypertension in intervention group versus proportion of controlled hypertension in control group at follow up. This is because the allocation to control versus intervention group is a randomized process and I would expect the proportions of controlled hypertensives in both trial arms to be essentially the same at baseline.
  It is important to have these statistical issues well thought out before data collection is completed.
11. Dissemination of findings: The authors should in the interests of open science consider having the data associated with the project made available to other researchers to reanalyze and build on their work. This can be through managed access or fully open processes.

Is the study design appropriate for the research question?

Yes

Is the rationale for, and objectives of, the study clearly described?

Yes

Are sufficient details of the methods provided to allow replication by others?

Yes

Are the datasets clearly presented in a useable and accessible format?

Not applicable

Reviewer Expertise:

Epidemiology of hypertension and other non-communicable diseases in Africa

I confirm that I have read this submission and believe that I have an appropriate level of expertise to confirm that it is of an acceptable scientific standard.
